# Age-related changes in EEG signal using triple correlation values

**DOI:** 10.3389/fnhum.2024.1438924

**Published:** 2024-09-25

**Authors:** Yuri Watanabe, Takashi Shibata, Mieko Tanaka, Kenji Ishii, Yuko Higuchi, Yohei Kobayashi, Yukio Kosugi

**Affiliations:** ^1^Brain Functions Laboratory, Inc., Tokyo, Japan; ^2^Department of Neurosurgery, Toyama University Hospital, Toyama, Japan; ^3^Department of Neurosurgery, Toyama Nishi General Hospital, Toyama, Japan; ^4^Tokyo Metropolitan Institute for Geriatrics and Gerontology, Tokyo, Japan; ^5^Department of Neuropsychiatry, University of Toyama Graduate School of Medicine and Pharmaceutical Sciences, Toyama, Japan; ^6^Research Center for Idling Brain Science, University of Toyama, Toyama, Japan

**Keywords:** EEG, correlation, dipole, aging, alpha-bands

## Abstract

The alpha rhythm in human electroencephalography (EEG) is known to decrease in frequency with age. Previous study has shown that elderly individuals with dementia exhibit higher S values (spatial variability) and SD values (temporal variability) in the triple correlation of the occipital region (P3, P4, Oz) compared to healthy elderly individuals. The objective of this research is to examine changes in S and SD values of the alpha band with aging in healthy individuals using triple correlation values from the frontal region. The subjects were 50 healthy elderly subjects (mean age 73.0 ± 5.1 years), 34 healthy younger subjects (mean age 28.1 ± 4.6 years), and 21 dementia patients (mean age 70.1 ± 9.1 years). The methodology involved recording EEG for 5 min during rest with closed eyes, and then calculating S and SD values of the alpha band (8-13 Hz) using three electrodes in the frontal region (F3, F4, Fpz). The findings indicated that the S values of young individuals were significantly higher than those of elderly individuals (*p* < 0.01), whereas the SD values of young individuals tended to be lower than those of elderly individuals. The elevated S values in young individuals imply greater spatial variability akin to individuals with dementia, whereas the reduced SD values in young individuals suggest lower temporal variability unlike individuals with dementia. The discrepancy between the S value and SD value in healthy young individuals suggests that the normal cortical dipole in the frontal regions might be more abundant in them compared to healthy elderly individuals.

## 1 Introduction

It’s generally known that the frequency of alpha waves decreases with age, while theta waves increase (Babiloni et al., 2006; Scally et al., 2018; Clements et al., 2021; Tröndle et al., 2023; Manor et al., 2023). Aging constitutes a significant risk factor for neurodegenerative diseases, and patients with dementia exhibit early electroencephalography (EEG) abnormalities as the condition progresses, contrasting with healthy elderly individuals. Consequently, quantitative assessment of EEG proves effective in discerning between normal and pathological brain aging (Rossini et al., 2013; Zappasodi et al., 2015). EEG changes due to aging and EEG changes due to dementia can be difficult to distinguish by changes in alpha wave power alone. This is because the characteristic decrease in alpha waves with aging is also observed in patients with dementia. Therefore, it is necessary to distinguish between changes in alpha waves due to aging and changes in alpha waves due to dementia. Our method of calculating the triple correlation value in our previous study (Watanabe et al., 2017; Watanabe et al., 2019) showed that it is possible to distinguish between elderly healthy subjects and dementia patients. EEG’s triple correlation values serve as an analytical tool for assessing abnormalities in cortical dipoles, estimated from three electrodes, by measuring spatial (S value) and temporal (SD value) variations. Our previous findings indicate that utilizing the S and SD values of triple correlation in the occipital region allows differentiation between healthy elderly individuals and those with dementia. Specifically, individuals with dementia display higher S and SD values in the theta to alpha band (6–13 Hz) in the occipital region (P3, P4, Oz) compared to their healthy counterparts, suggesting cortical dipole abnormalities in that frequency range. However, as described in Supplementary material, there was no statistically significant difference in S values when comparing healthy older subjects and healthy young subjects. With this method, it was difficult to distinguish between healthy older subjects and healthy young subjects. Therefore, we investigated the optimal electrode and frequency band to distinguish between healthy young subjects, healthy elderly subjects, and dementia patients by using a method based on triple correlation values that are not affected by the decrease in power values due to aging.

It is important to distinguish between age-related decline in brain function in healthy individuals and decline in brain function due to dementia. Although some previous studies have mentioned changes in EEG with aging, no study has quantitatively evaluated the difference between aging-related changes and dementia using EEG with a small number of electrodes. Thus, gaining insight into the generation and alteration of cortical dipoles during normal aging is imperative for a deeper understanding of dementia pathology.

Typically, dipole estimation methods like the current dipole method and current source distribution estimation capture intense local synchronous neuronal activities such as epileptic potentials and evoked potentials to identify potential sources (He et al., 1987; Ebersole and Hawes-Ebersole, 2007). However, traditional dipole estimation methods focus solely on strong synchrony, hindering the analysis of random or asynchronous neural activities—a limitation that complicates their application to the highly variable alpha rhythm. Conversely, EEG triple correlation, unlike traditional dipole estimation, excels in quantifying spatiotemporal variations, encompassing weak synchrony, random activity, and strong synchrony, albeit without pinpointing the dipole’s source of generation. Hence, in this study, we opted to explore the differences between healthy young and elderly individuals using triple correlation values to grasp the intricacies of the highly variable alpha band.

## 2 Method

### 2.1 Datasets used to evaluate the proposed method

EEG data were recorded with resting closed eyes and body motionless. Data from two medical institutions were used to evaluate the proposed methodology.

Data on healthy young subjects were obtained from 34(male: 21, female: 13) healthy subjects (mean age 28.1 years ±4.6) at the University of Toyama Hospital. EEG recordings were obtained with a Nihon Kohden EEG device (EEG-1250 version 07–02, Nihon Kohden Corp.) and a 32-channel MCS cap (Medical Computer Systems Ltd.) in a wave-shielded and sound-attenuated room. Data were collected with a sampling rate of 500 Hz. The bandwidth was set at 0.53–120 Hz with a 60 Hz notch filter. The study was conducted in accordance with the Declaration of Helsinki. The Committee on Medical Ethics of Toyama University approved the present study (No. I2013006). After providing a full explanation of the purpose and procedures of the study, written informed consent was obtained individually from each study participant. For participants under 20 years of age, written consent was also received from a parent or guardian.

Data on healthy elderly subjects and dementia patients were obtained at the Tokyo Metropolitan Institute for Geriatrics and Gerontology. The elderly healthy 50(male: 9, female: 41) subjects (mean age 73.0 years ±5.1) were selected from elderly subjects who volunteered to undergo annual FDG-PET, MRI and neuropsychological examinations (PET examination showing brain activity using [F-18]-labeled fluorodeoxyglucose). Among them, those who exhibited good PET results (rated A out of grades A, B, and C) with normal MRI and neuropsychological tests were classified as the normal control group. For the diagnostic criteria of AD (McKhann et al., 2011), 21 (male: 4, female: 17) patients (mean age 70.1 years ±9.1) who met the criteria for probable AD dementia in the core clinical criteria and whose FDG-PET findings were consistent with the diagnosis of AD were selected as the AD group. The filter during recording ranged from 0.08 to 300 Hz, and 21 electrodes were placed according to the International 10–20 system. This study was approved by the ethics committee of Tokyo Metropolitan Institute for Geriatrics and Gerontology and EEG examinations were performed on those who gave consent and wished to have them.

For this analysis, we used data from only three electrodes with a reference electrode placed on the right earlobe. The sampling frequency at the time of analysis was 200 Hz.

### 2.2 Triple correlation

The conceptual basis of the triple correlation value lies in its application to the estimation of seismic waves. Specifically, seismic waves show significant variations between observation points when the epicenter is near the Earth’s surface. Conversely, if the epicenter is deep underground, a type of seismic wave known as a P-wave maintains consistent amplitude and phase across all locations. Similarly, when estimating the dipoles that are the source of brain signals, triangular regions formed by three electrodes are used. Analyzing EEG data recorded from these electrodes does not allow for the precise identification of the exact locations of deep brain virtual dipole sources. However, it does provide rough estimates of the spatiotemporal changes associated with these virtual dipole sources.

Therefore, we defined a triple correlation value $S_{t}$ using the product of the time series EVA(t), EVB(t), and EVC(t) of brain potentials observed from a set of three electrodes among 21 electrodes in accordance with the international 10–20 method, and the signals with time deviations of τ1 and τ2 from the potential signal of one electrode (Watanabe et al., 2017).

The calculation method of the triple correlation values is explained in detail below (Equations 1–3). First, define a function *θ*(x,y,z) that takes real numbers x, y, and z as arguments and takes the value 1 when (x > 0, y > 0, z > 0) or (x < 0, y < 0, z < 0) and 0 otherwise.


(1) $$\begin{array}{l} \theta \left(x,y,z\right)\\ \left\{ \;\begin{array}{c} 1,\left(x>0\;\mathrm{and}\;y>0\;\mathrm{and}\;z>0\right)\;\mathrm{or}\;\left(x<0\;\mathrm{and}\;y<0\;\mathrm{and}\;z<0\right)\\ 0,\left(\mathrm{otherwise}\right) \end{array}\right. \end{array}$$

When time is t and the time series of brain potentials recorded at the three locations are EVA(t), EVB(t), and EVC(t), the triple correlation value can be expressed by the following equation.


(2) $$\begin{array}{l} S_{t}\left(\tau_{1},\tau_{2}\right)=\\ \frac{1}{N_{t}}\overset{M-1}{\underset{m=0}{\sum }}|\begin{array}{c} \theta \left(EVA\left(t+m\Delta t\right),\;EVB\left(t+m\Delta t\right),\;EVC\left(t+m\Delta t\right)\right)\\ \times EVA\left(t+m\Delta t\right)EVB\left(t+m\Delta t-\tau_{1}\right)EVC\left(t+m\Delta t-\tau_{2}\right) \end{array}| \end{array}$$

(3) $$N_{t}=\overset{M-1}{\underset{m=0}{\sum }}\theta \left(EVA\left(t+m\Delta t\right),\;EVB\left(t+m\Delta t\right),\;EVC\left(t+m\Delta t\right)\right)$$

where τ_1 and τ_2 are delay times, Δt = 0.005 s is the sampling interval, M = 200 since the interval for calculating the triple correlation value is 1 s and the sampling frequency is 200 Hz. *N_t_* is the number of sample points in the analysis interval (m = 0.M-1) where the three EEG signals have the same sign.

In a previous study, three electrodes in the occipital region (P3, P4, Oz) were used in the bandwidth of from 6 to 13 Hz to discriminate dementia patients from elderly healthy subjects. Two biomarker S and SD values are calculated from the calculated triple correlation values; the S value indicates the spatial variation of the triple correlation values and the SD value indicates the temporal variation of the triple correlation values. The calculation methods of S and SD values are described in Supplementary material. In order to find the optimal electrode, a linear discriminant analysis was conducted using S and SD values for all 12 combinations of 3 electrodes in the frontal, temporal, occipital, and parietal regions. As a result, the three electrodes (P3, P4, and Oz) on the occipital area were selected because they showed significant differences in both S and SD values (see Supplementary material) and had the highest percentage of correct responses.

Both S and SD values of dementia patients were greater than those of healthy elderly subjects, thus it is possible to separate dementia patients from elderly healthy subjects. However, there was no difference in S values between healthy youth and healthy elderly, with healthy youth having the smallest SD values and dementia having the largest SD values. This result shows in Supplementary material.

Since the SD value is calculated from the time direction variation of the triple correlation value, the SD value is larger when the waxing and waning of the three electrode potentials is disturbed. It is also known that the lower the mean frequency, the larger the SD value (Watanabe et al., 2017). Due to the effects of aging and slow wave transformation, SD values are larger in healthy elderly persons and dementia patients than in healthy young persons. However, as mentioned above, the S value cannot distinguish between healthy young and healthy elderly.

Our current objective is to distinguish between healthy young individuals and healthy elderly individuals. To achieve this, we examined suitable electrodes and frequency bands and have chosen to focus on triple correlation values measured via frontal electrodes (F3, F4, and Fpz) within the alpha band (8–13 Hz) for this study. Next, we investigated differences between the left and right hemispheres of the frontal region in healthy young and elderly individuals based on the triple correlation values among the electrodes placed on the left forehead (F3, Fpz, and Cz) and right forehead (F4, Fpz, and Cz). Finally, we examined how the triple correlation values change depending on the areas covered by the three selected electrodes: (F8, Fpz, and Cz) and (F7, Fpz, and Cz) as large regions, (F4, Fpz, and Cz) and (F3, Fpz, and Cz) as medium regions, and (F4, Fp2, and Fz) and (F3, Fp1, and Fz) as small regions. Through this analysis, we explored the characteristics of the S value and SD value associated with these triple correlation values.

In order to compare differences among young healthy subjects, elderly healthy subjects and dementia patients, statistical analyses were performed with the Kruskal-Wallis test followed by the Steel-Dwass test.

## 3 Result

### 3.1 Triple correlation value at the frontal area

**Figure 1:**
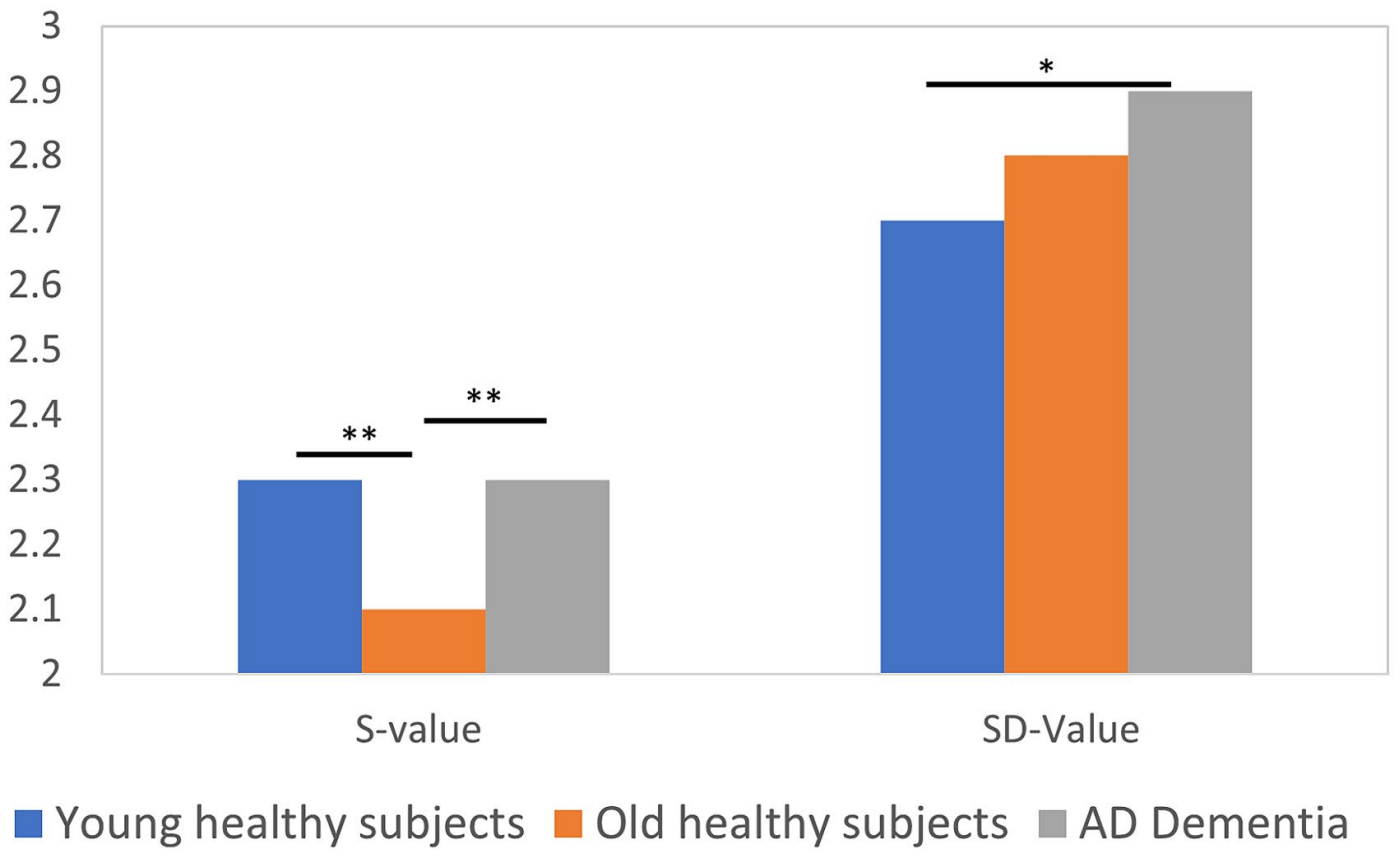
The triple correlation Value for F3F4Fpz (8-13 Hz).

Triple correlation values were calculated in the forehead (F3, F4, and Fpz) in the alpha band (8–13 Hz) and the results are shown in Figure 1. Young healthy subjects had larger S values and smaller SD values than older healthy subjects. Kruskal-Wallis test showed that there was a statistically significant difference (*p* < 0.01) in S values between young healthy subjects and elderly healthy subjects, and between elderly healthy subjects and dementia patients. In addition, there was a statistically significant difference in SD values between young healthy subjects and dementia patients (*p* < 0.05). The effect size and power of the test for the combinations with statistically significant differences in the *p*-values mentioned above were examined, and the results were as follows: effect size = 0.92 and power = 0.98 for young healthy subjects and elderly healthy subjects with S values, effect size = 0.85 and power = 0.97 for elderly healthy subjects with S values and dementia patients, and effect size = 0.76 and power = 0.82 for young healthy subjects with SD values and dementia patients, all of which confirm the appropriateness of the sample sizes. The effect size was 0.85, power of the test = 0.97. Furthermore, the effect size for young healthy subjects and dementia patients with SD value = 0.76 and power of the test = 0.82. These results confirmed that the sample size was appropriate.

### 3.2 Left–right difference in triple correlation values

**Figure 2:**
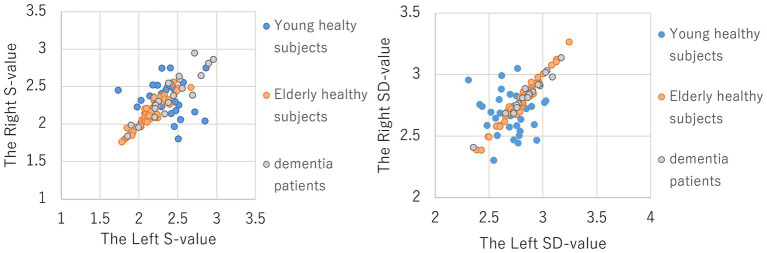
Distribution of left–right differences in S and SD values in young healthy subjects, elderly healthy subjects and dementia patients (Left figure: S-Value, Right figure: SD-Value).

To examine whether there is a left–right difference in the triple correlation values between healthy elderly, healthy young subjects, and dementia patients, S and SD values were calculated using the left forehead (F3, Fpz, and Cz) and right forehead (F4, Fpz, and Cz). As a result, as shown in Figure 2, there were variations in the left and right S values and SD values in young healthy subjects, but the left–right differences were small in elderly healthy subjects and dementia patients. To analyze the left–right differences quantitatively, multiple comparisons were performed by squaring the difference values of the left–right S and SD values, respectively; S values showed statistically significant differences (*p* < 0.01) between all groups, while SD values showed statistically significant differences (p < 0.01) between healthy elderly and healthy young subjects, and between young healthy subjects and dementia patients, but no statistically significant difference was found between the elderly healthy subjects and dementia patients. This is also consistent with the previously mentioned small difference in SD values between elderly healthy subjects and dementia patients at the frontal electrodes (F3, F4, and Fpz). This indicates that bipolar activity is more pronounced in the young compared to the elderly, and that with aging, the left–right difference tends to become smaller in the healthy elderly as well as in dementia patients.

### 3.3 Evaluation of different areas for calculating triple correlation values

**Figure 3:**
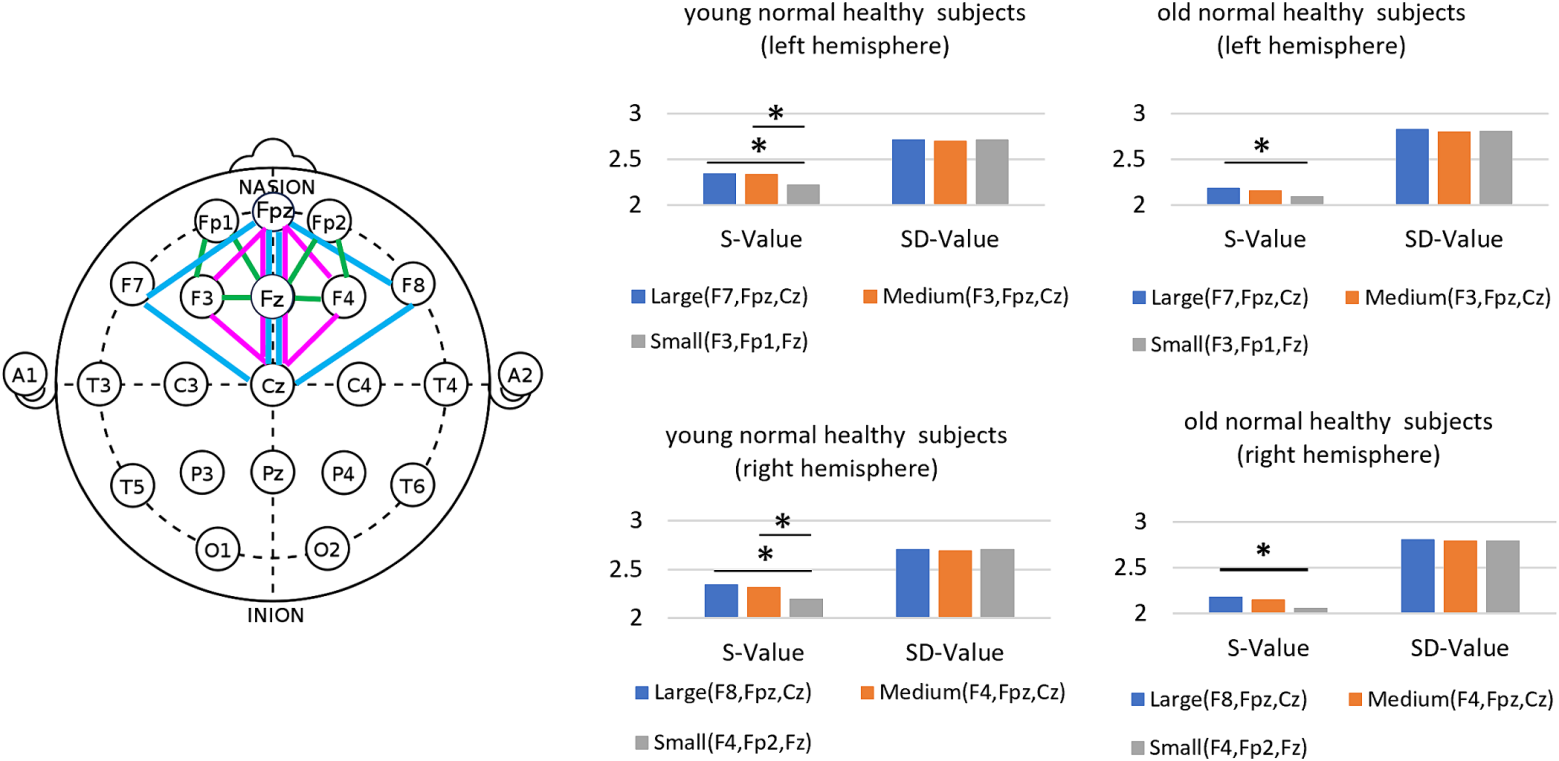
Areas to calculate the triple correlation values and results of S-Value and SD-value.

We examined how the S and SD values changed with variations in the area of the region where triple correlation values were calculated, categorized as large, medium, and small. As depicted in Figure 3, we defined (F8, Fpz, and Cz) and (F7, Fpz, and Cz) as large regions, (F4, Fpz, and Cz) and (F3, Fpz, and Cz) as medium regions, and (F4, Fp2, and Fz) and (F3, Fp1, and Fz) as small regions. As the area of the three electrodes decreased, the S values decreased for both older and younger subjects, decreasing by 5% compared to the larger area case. In contrast, the SD value remained largely unchanged despite variations in the area of the three electrodes. Similarly, the mean frequency also remained nearly constant as the area of the three electrodes changed. We interpret this to mean that in smaller areas, the S value decreases due to the fewer number of dipoles, resulting in less variation in the triple correlation value. Conversely, when the area of the triangle is enlarged, multiple dipoles with larger variations are encompassed within the region.

## 4 Discussion

**Figure 4:**
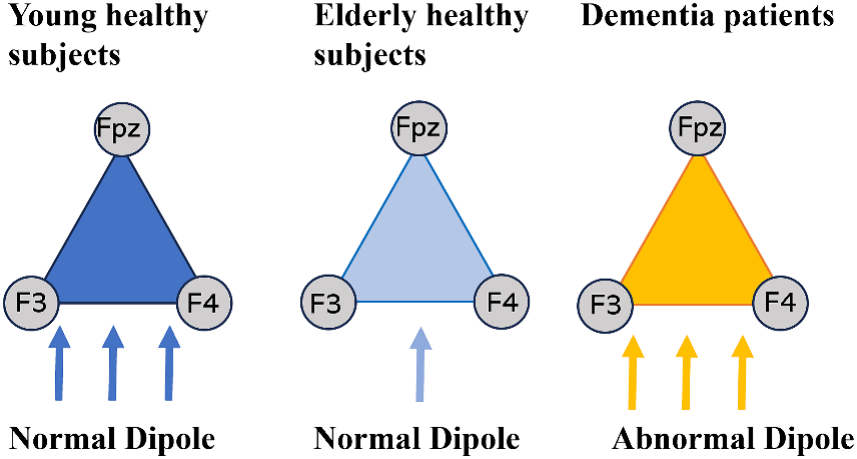
Three types of dipoles.

The findings of this study revealed that the spatial dispersion (S value) of young, healthy individuals is comparable to that of dementia patients. Initially, this posed a challenge as S values alone could not differentiate between young, healthy individuals and elderly dementia patients. To address this, we introduced three types of dipoles, as illustrated in Figure 4, which enabled a more effective interpretation of the results. We labeled the dipoles of young, healthy individuals as “young dipoles,” those of elderly, healthy individuals as “aging dipoles,” and those of dementia patients as “abnormal dipoles.

Based on our analysis, “young dipoles” have demonstrated higher spatial dispersion in S values and lower temporal dispersion in SD values compared to “aging dipoles.” This implies the likelihood of a more frequent generation of stable “young dipoles” within the frontal cortex. On the other hand, “abnormal dipoles” in dementia not only demonstrate high spatial variability (S value) but also high temporal variability (SD value), allowing for differentiation from young normal dipoles with small temporal dispersion (SD value) in triple correlation values. In summary, both young dipoles and abnormal dipoles in dementia exhibit significant spatial variability in triple correlation values due to the generation of multiple dipoles within the cerebral cortex but incorporating temporal dispersion (SD value) enables quantification of the spatiotemporal variation of dipoles. While alpha waves in dementia patients are known to have slow frequencies without waxing and waning, whether the SD value of abnormal dipoles reflects alpha wave characteristics such as waxing and waning, continuity, and rhythmicity is a topic for future research.

In our investigation, there was no difference in the triple correlation value S value in the occipital region between healthy elderly and young individuals. The alpha band has often been used in previous studies because it appears primarily in the occipital region (Lizio et al., 2011). However, spectral analysis focusing on power values posed a challenge in distinguishing whether differences between healthy young and elderly individuals are solely due to normal aging or pathological decline in cognitive function. Nevertheless, in this study, using triple correlation values in the frontal region instead of the occipital region suggested the possibility of identifying normal aging changes. In other words, our finding that the characteristics of early aging in healthy subjects are more likely to appear in the frontal area than in the occipital area corresponds well with the age-related decline in frontal lobe function (Ishibashi et al., 2018) noted in brain imaging and psychological assessment. This implies that occipital lobe function is maintained with aging, which is also consistent with the fact that no difference was found between the elderly and the young in S values in the occipital region.

And it suggests that capturing EEG changes including not only S values but also SD values in the frontal area is effective for early detection of normal aging and suspected dementia. Findings from NAT analysis using EEG suggest that the complexity of EEG signals increases from younger to middle-aged adults and decreases with age after adulthood (Ishii et al., 2017), but this study did not include EEG data from middle-aged individuals (around their 40s). Additionally, it remains unclear how abnormal dipoles in dementia, such as those in Alzheimer’s disease, Lewy body dementia, vascular dementia, and other mental disorders (e.g., depression), differ. Hence, further research is necessary to understand the limitations and effectiveness of EEG triple correlation values.

This study investigated alpha rhythm changes between young and elderly individuals using EEG resting recordings. The results showed that young individuals exhibited higher spatial variability (S values) and lower temporal variability (SD values) compared to elderly individuals. Notably, this high spatial variability (S value) resembles the characteristics observed in dementia. These findings suggest the potential presence of a stronger normal cortical dipole in the frontal regions of healthy young individuals compared to healthy elderly individuals.

In addition, EEG is not only less expensive and simpler than PET or MRI, but this method can be analyzed using only three electrodes, making it a simple and easy system to use. Therefore, it is possible to analyze the EEG obtained using a simple electroencephalograph capable of measuring 3-electrode EEG. In the future, we believe that this system can be applied not only in medical institutions but also in companies and visiting nurses, where EEG can be simply measured, and the data analyzed on a server and fed back to the subject.

## Data Availability

The original contributions presented in the study are included in the article/Supplementary material, further inquiries can be directed to the corresponding author/s.
