# Supplementary material for: Age-related changes in EEG signal using triple correlation values
Source: Front Hum Neurosci. 2024 Sep 25;18:1438924. doi: 10.3389/fnhum.2024.1438924 (PMC11461205; doi:10.3389/fnhum.2024.1438924)
Supplement: Supplementary file 1 [file Data_Sheet_1.docx]

Supplementary Material

Age-related changes in EEG signal
using triple correlation values

Yuri Watanabe^1^, Takashi Shibata^2,3^, Mieko Tanaka^1^, Kenji Ishii^4^, Yuko Higuchi^5,6^ ,

Yohei Kobayashi^1^, Yukio Kosugi^1^

^1^Brain Functions Laboratory, Inc., Tokyo, Japan

^2^Department of Neurosurgery, Toyama University Hospital, Japan

^3^Department of Neurosurgery, Toyama Nishi General Hospital, Japan

^4^Tokyo Metropolitan Institute of Gerontology, Tokyo, Japan

^5^Department of Neuropsychiatry, University of Toyama Graduate School of Medicine and Pharmaceutical Sciences, Toyama, Japan

^6^Research Center for Idling Brain Science, University of Toyama, Toyama, Japan

*** Correspondence: Yuri Watanabe: ywatanabe@bfl.co.jp**

# Calculation method of S and SD values


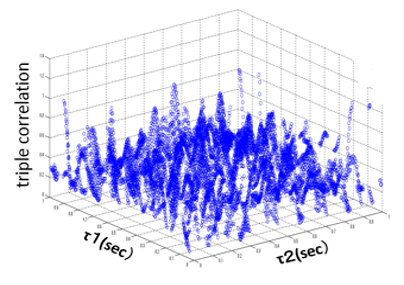
If the calculation of the triple correlation value calculation is performed every second, the triple correlation value is plotted on the feature space formed by the two delay parameters τ_1_，τ_2_ as shown in Figure 1. The triple correlation value is compared to a forest of trees. The height of the tree forest (the maximum value of the triple correlation value) is the triple correlation value, and the interval where the tree forest is planted (the interval between τ_1_ and τ_2_ where the triple correlation value takes the maximum value) is the interval between adjacent time points, and the S value of the degree of variation of the triple correlation value and the SD value of the degree of variation between τ_1_ and τ_2_ where the triple correlation value takes the maximum value were evaluated.

**Figure 1.** The distribution of triple correlation values

| Data | healthy young subjects | healthy elderly subjects | dementia patients |
| --- | --- | --- | --- |
| N(M/F) | 34(21/13) | 50(9/41) | 21(4/17) |
| Age(y) | 28.1 ±4.6 | 73.0 ±5.1 | 70.1 ±9.1 |

# Datasets

Details of the data sets used in the analysis are as follows

# Results for occipital lobe

# Figure 2 shows the results of S values, SD values, and mean frequency when triple correlation values were calculated at 6-13 Hz using the occipital lobes (P3, P4, Oz). The mean frequency was calculated in the band of 6-13 Hz.

**Figure １.** The distribution of triple correlation values

# The parameter of triple correlation and results.

EEGs from 52 healthy subjects (age 71.9±5.9) and 20 dementia patients (84.4±6.3) at rest and closed eye were used and analyzed to find the optimal electrodes.A linear discriminant analysis was performed using S and SD values for a total of 12 3-electrode combinations on the frontal, temporal, occipital, and parietal regions of the head. As shown in Table 1, the three electrodes on the occipital area (P3, P4, and Oz) were selected because they had the highest percentage of correct responses and the p-values for both S and SD values, the explanatory variables, were statistically significant (p<0.05).

Table 1. The parameter of triple correlation and results.

| Three electrodes | S value (P value) | SD value (P value) | Accuracy |
| --- | --- | --- | --- |
| **P3P4Oz** | **0.01(P<0.05)** | **0.03(P<0.05)** | **75%** |
| F7T3T5 | 0.82(P>0.06) | **0.01(P<0.05)** | 70% |
| T3T5C3 | 0.89(P>0.05) | **0.02(P<0.05)** | 68% |
| F3F4Fpz | 0.90(P>0.05) | 0.06(P>0.05) | 67% |
| F8T6T4 | 0.64(P>0.05) | **0.001(P<0.01)** | 67% |
| T3T4Fpz | **0.02(P<0.05)** | 0.05(P>0.05) | 67% |
| P3P4Cz | 0.47(P>0.05) | **0.02(P<0.05)** | 65% |
| PzO1O2 | 0.79(P>0.05) | **0.02(P<0.05)** | 65% |
| T4T6C4 | 0.17(P>0.05) | 0.06(P>0.05) | 64% |
| T3T4Oz | 0.14(P>0.05) | **0.03(P<0.05)** | 63% |
| C3C4Pz | 0.09(P>0.05) | **0.02(P<0.05)** | 63% |
| F3F4Cz | 0.71(P>0.05) | **0.02(P<0.05)** | 63% |

The above results are presented in the following peer-reviewed papers already published.

Yuri Watanabe, Yohei Kobayashi, Mieko Tanaka, Satoshi Orimo, Takashi Asada and Tohru Yagi. （2019）, Analysis for Alzheimer’s Disease and Dementia with Lewy Bodies, Transactions of the Society of Instrument and Control Engineers (in Japanese), Vol.55, No.9, 536/544
